# Supplementary material for: Low temperature below 200 °C solution processed tunable flash memory device without tunneling and blocking layer
Source: Nat Commun. 2019 May 13;10:2143. doi: 10.1038/s41467-019-10142-y (PMC6514002; doi:10.1038/s41467-019-10142-y)
Supplement: Supplementary file 1 — Supplementary Information [file 41467_2019_10142_MOESM1_ESM.pdf]

Low temperature below 200°C solution processed tunable flash memory device without tunneling and blocking layer

Sandip Mondal,[1,2a] and V Venkataraman[1]

[1]*Department of Physics, Indian Institute of Science, Bangalore 560012, India and*

[2]*SanDisk (Western Digital Corporation) India Device Design Center, Bangalore 560103, India*

[a] Correspondence and requests for materials should be addressed to e-mail: S.M. (sandip@iisc.ac.in)

CONTENTS

Supplementary Note 1 3

Supplementary Note 2 4

Supplementary Note 3 5

Supplementary Note 4 8

Supplementary Note 5 9

Supplementary Note 6 10

Supplementary Note 7 11

Supplementary Note 8 12

Supplementary Note 9 13

Supplementary Note 10 15

SUPPLEMENTARY NOTE 1

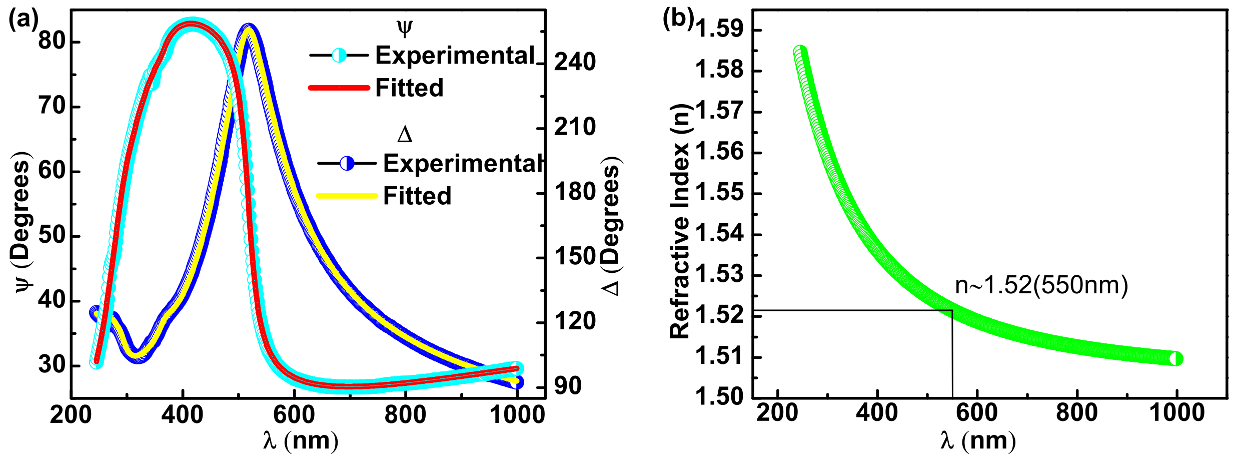

Supplementary Figure 1 | Ellipsometry characterization of ALPO thin film : (a) Experimental and fitted $\psi - \Delta$ with λ plot of ALPO thin film (139 nm) on Si substrate, annealed at 800°C. The measurements were performed with an incidence angle of 65° across the spectral wavelength range 250−1000 nm. Fitting $\psi - \Delta$ of the films with a Cauchy dispersion model by spectroscopic ellipsometry (SE) parameters enabled the estimation of refractive index (n) and thickness of the film. The absence of any inter-layer between Si and ALPO films is confirmed by ellipsometry analysis. (b) The refractive index as a function of wavelength extracted from ellipsometry. The observed refractive index is 1.52 at wavelength of 550 nm. The absorption coefficient is zero in this wavelength range. There is no change in refractive index with variation of annealing temperature.

SUPPLEMENTARY NOTE 2

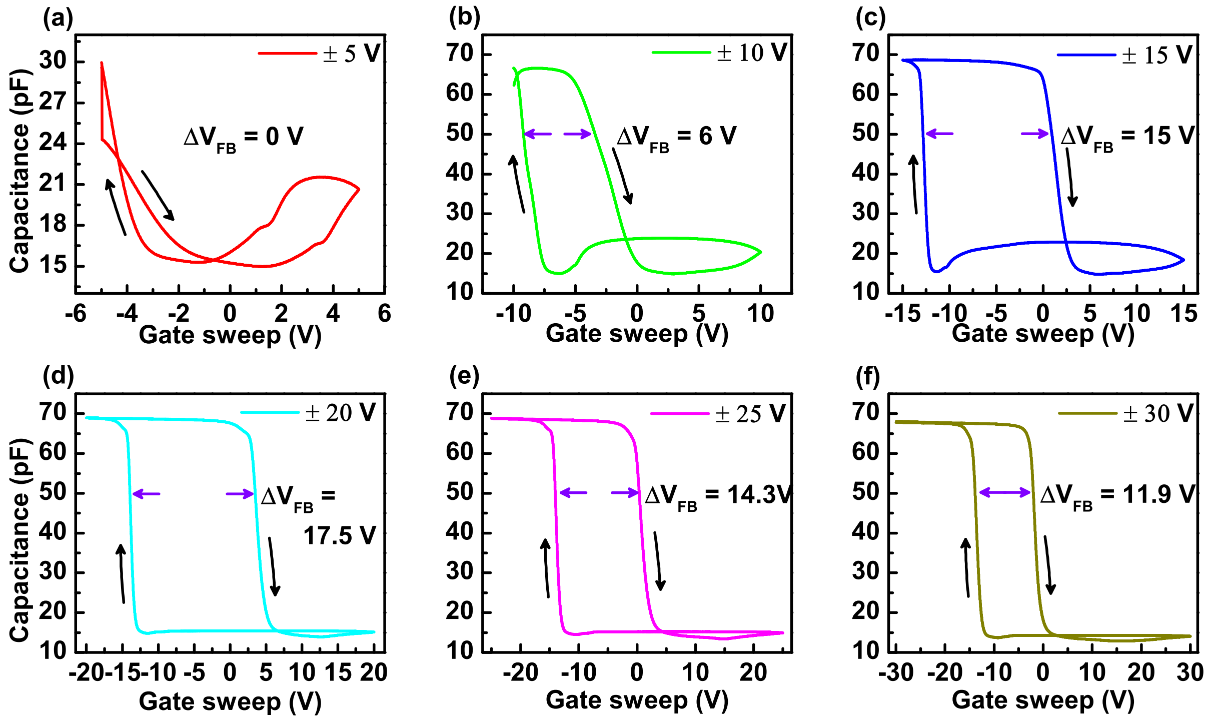

Supplementary Figure 2 | Flatband voltage variation with respect to sweep voltage (ΔV_{FB}) on gate sweep voltage. The arrow (black) indicates the direction of sweep voltage. The highest ΔV_{FB} observed is 17.5 V at gate sweep voltage of ± 20 V. Film thickness is 139 nm and annealing temperature is 200°C.

SUPPLEMENTARY NOTE 3

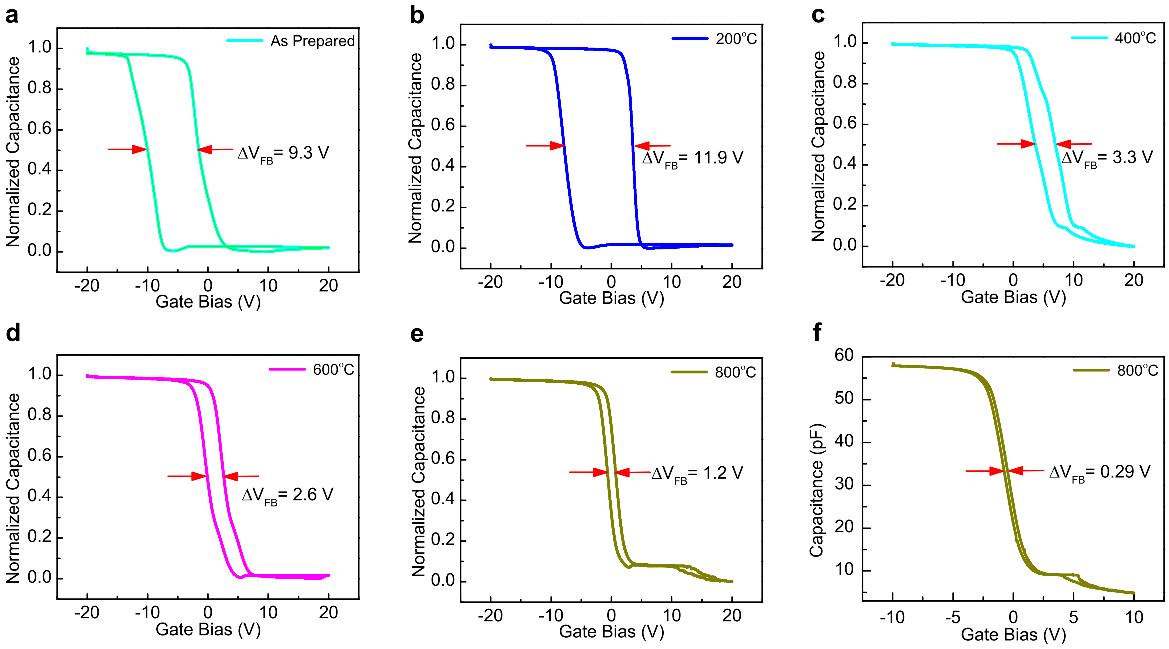

Supplementary Figure 3a | Intrinsic defects in ALPO : Films ($\sim$ 91 nm) annealed at different temperatures were tested for electrical defects by CV measurements. A large flatband voltage window (ΔV_{FB}) was observed for the low temperature annealed ALPO film(below 400°C). Fig. a, b, c, d and e illustrate the hysteresis for As Prepared, 200°C, 400°C, 600°C and 800°C annealed (for 1 hour) films, respectively. The voltage was swept from -20V to $+20$V and back with a rate of 2V/min. It is also observed that the trap charges in the ALPO decreases with the decrease of sweep voltage (Fig. f, Sweep voltage -10V to $+10$V). The statistics of the trap charges in ALPO is shown in the TABLE.

Supplementary Table - 1 : Defect analysis of ALPO

Annealed Temperature(°C)	V_{FB}(V)	Cox/Unit Area(F/cm^2)	Q_{ot}(cm^{-2})	Figure
As Prepared	9.3	4.7×10^{-8}	2.7×10^{12}	a
200	11.9	4.7×10^{-8}	3.5×10^{12}	b
400	3.3	5.5×10^{-8}	1.1×10^{12}	c
600	2.6	6.1×10^{-7}	9.9×10^{12}	d
800	1.2	8.5×10^{-8}	6.7×10^{11}	e
800	0.29	8.5×10^{-8}	1.3×10^{11}(SV=$\pm$10V)	f

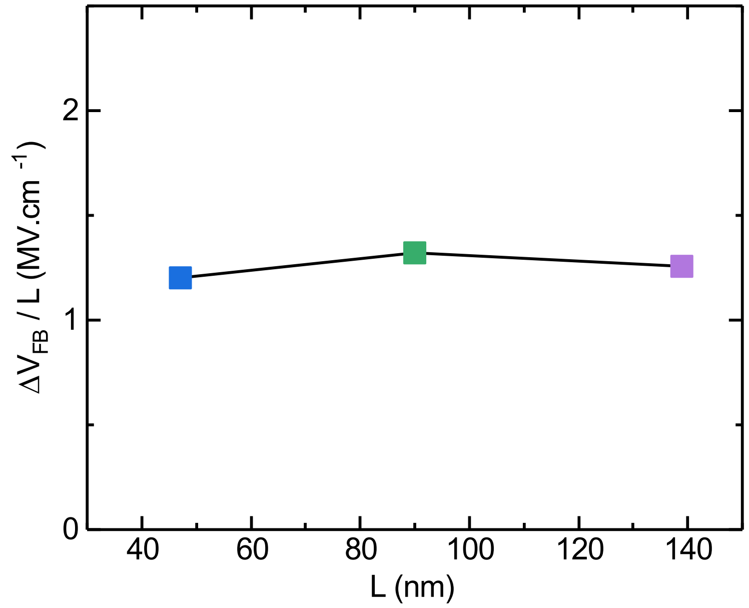

Supplementary Figure 3b | Hysteresis window with respect to thickness: The memory device fabricated with the thickness of ALPO of 47, 91 and 139 nm respectively. All the devices were annealed at 200°C.

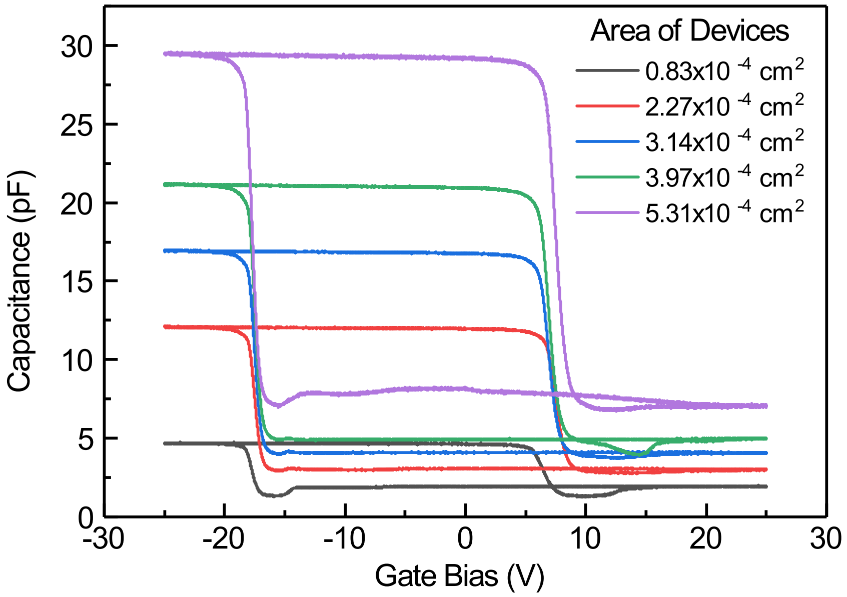

Supplementary Figure 3c | Hysteresis window with respect to device dimension: The devices have been fabricated with different area (0.83×10^{-4} cm^2, 2.27×10^{-4} cm^2, 3.14×10^{-4} cm^2, 3.97×10^{-4} cm^2 and 5.31×10^{-4} cm^2) with constant thickness of AP-ALPO ($\sim$ 130 nm). The memory response appears similar in all cases indicating operational scheme at lower device dimension.

SUPPLEMENTARY NOTE 4

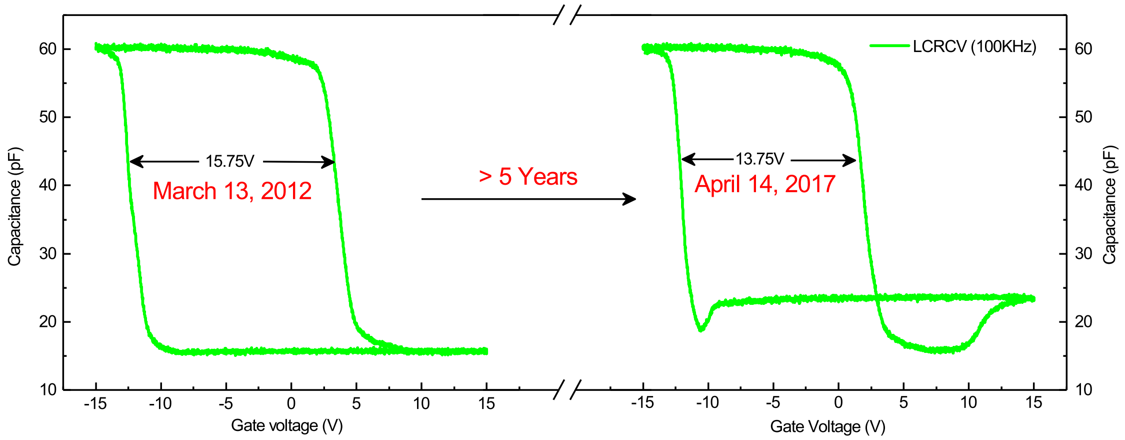

Supplementary Figure 4 | A strong memory property of AP-ALPO: The CV hysteresis curve for ALPO (AS Prepared) MOS device. Negligible degradation of CV hysteresis is observed after 5 years in open normal lab environment.

SUPPLEMENTARY NOTE 5

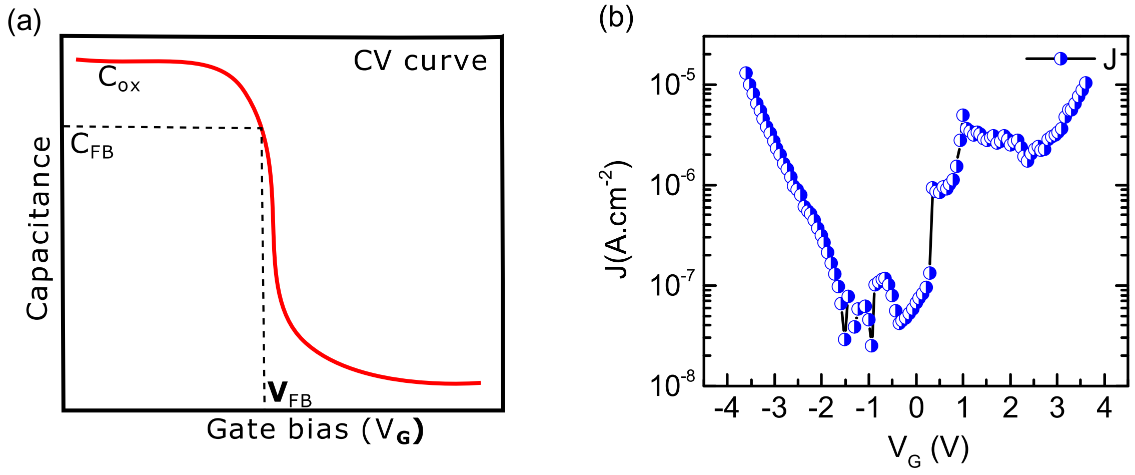

Supplementary Figure 5 | Flatband and leakage current property of devices: (a) The flatband voltage has been calculated from flatband capacitance (C_{FB}) [Nicollian, E. H., & Brews, J. R. (1981), MOS (metal oxide semiconductor) physics and technology, New York, Wiley]

$$C_{FB} = \frac{C_{ox}C_{sFB}}{C_{ox} + C_{sFB}} \tag{S−Eq1}$$

where, C_{sFB} is the semiconductor surface capacitor and C_{ox} is the total oxide capacitance.

The value of the C_{sFB} is calculated from,

$$C_{sFB} = \frac{\epsilon_s.\epsilon_0 A}{L_D} \tag{S−Eq2}$$

where, ϵ_s= Relative permittivity of semiconductor, ϵ_0= Permittivity of vacuum [F.cm^{-1}]. L_D= Debye length [cm] and A = Gate area of the capacitive devices [cm^2].

The Debye length is found from,

$$L_D = \sqrt{\frac{kT\epsilon_s\epsilon_0}{q^2 N_D}} \tag{S−Eq3}$$

where, k= Boltzmann constant [J.K^{-1}]. T= Temperature [K], q= Electronic charge [C], N_D = Doping concentration of the semiconductor [cm^{-3}].

The doping of the semiconductor is calculated from the slope of linear region of the (1/C^2)–V plot with the formula,

$$N_D = \frac{2}{q\epsilon_s.\epsilon_0.|slope|. \times A^2} \tag{S−Eq4}$$

(b) The leakage current density (J) as a function of electric field (E) has been measured for 139 nm ALPO film annealed at 200°C for one hr. The measured J at -1 MV cm^{-1} is -4.5×10^{-8} A cm^{-2}

SUPPLEMENTARY NOTE 6

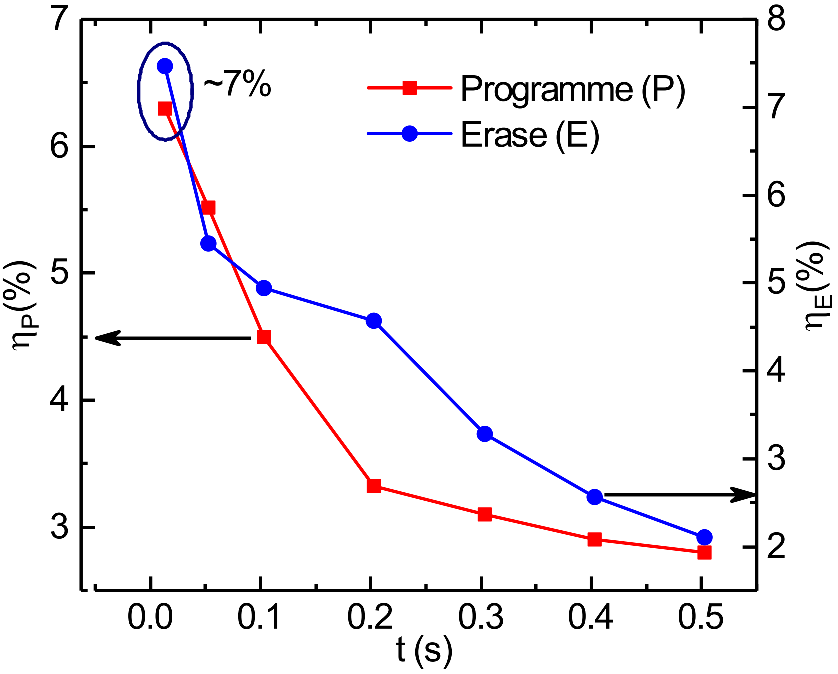

Supplementary Figure 6 | Charge Capturing efficiency of ALPO annealed at 200°C: The charge capturing efficiency has been calculated during programming and erasing. The highest capturing efficiency has been found to be 7% at 33V with 10ms P/E pulses

SUPPLEMENTARY NOTE 7

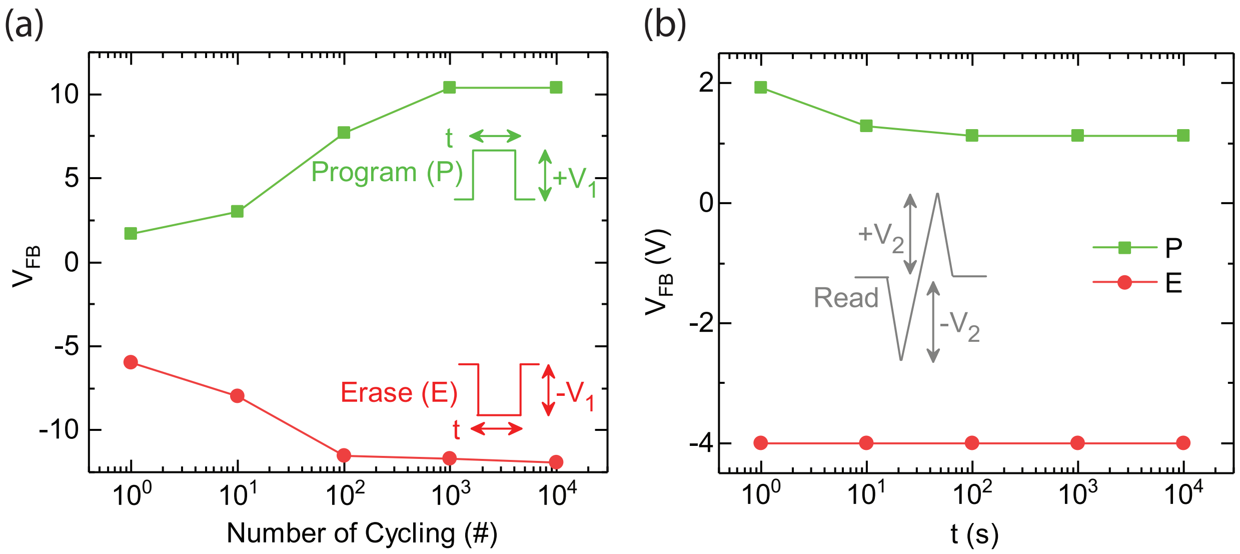

Supplementary Figure 7 | Endurance and Retention characteristics of device with ALPO annealed at 200°C: (a)Endurance characteristics of devices upto 10000 cycle. (b) Retention characteristics of the devices till 10000s

SUPPLEMENTARY NOTE 8

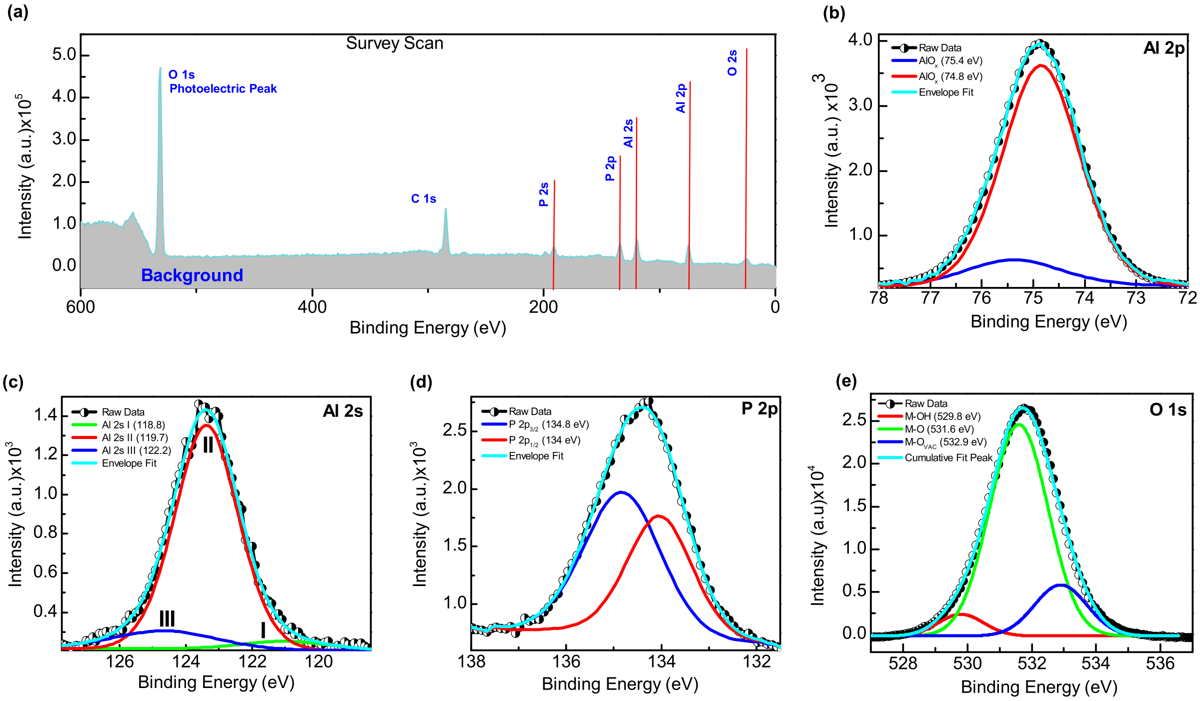

Supplementary Figure 8 | Material characterization of ALPO: (a) The XPS survey scan spectrum of ALPO annealed at 200°C (for 1 hour) by using 180 eV pass energy with a step 1eV. The survey scan shows all the elements present in ALPO (with P/Al $\sim$ 0.5). The reference C 1s peak is at 284.6 eV. (b) The high resolution Al 2p peak is de-convoluted into peaks corresponding to AlO_x (position at 74.8 eV, concentration of 87.20% and FWHM 1.76 eV) and AlO_x (position at 75.4 eV, concentration of 12.80% with FWHM 2.18 eV), (c) Al 2s components are Al 2s I (position at 120.8 eV, concentration of 18.31% with FWHM 4.62 eV), Al 2s II (position at 123.4 eV, concentration of 78.96% with FWHM 2.14 eV) and Al 2s III (position at 124.6 eV, concentration of 2.73% with FWHM 2.24 eV), (d) P 2p components are $2p_{3/2}$ (position at 134.8 eV, concentration of 57.51% with FWHM 1.90 eV) and P $2p_{1/2}$ (position at 134 eV, concentration of 42.49% with FWHM 1.62 eV), (e) O 1s components are M$-$O (position at 529.8eV, concentration of 5.9% with FWHM 1.7 eV), M$-$OH (position at 531.6 eV, concentration of 74.3% with FWHM 2.1eV) and M$-O_{VAC}$ (position at 532.9eV, concentration of 16.1% with FWHM 1.9eV)

SUPPLEMENTARY NOTE 9

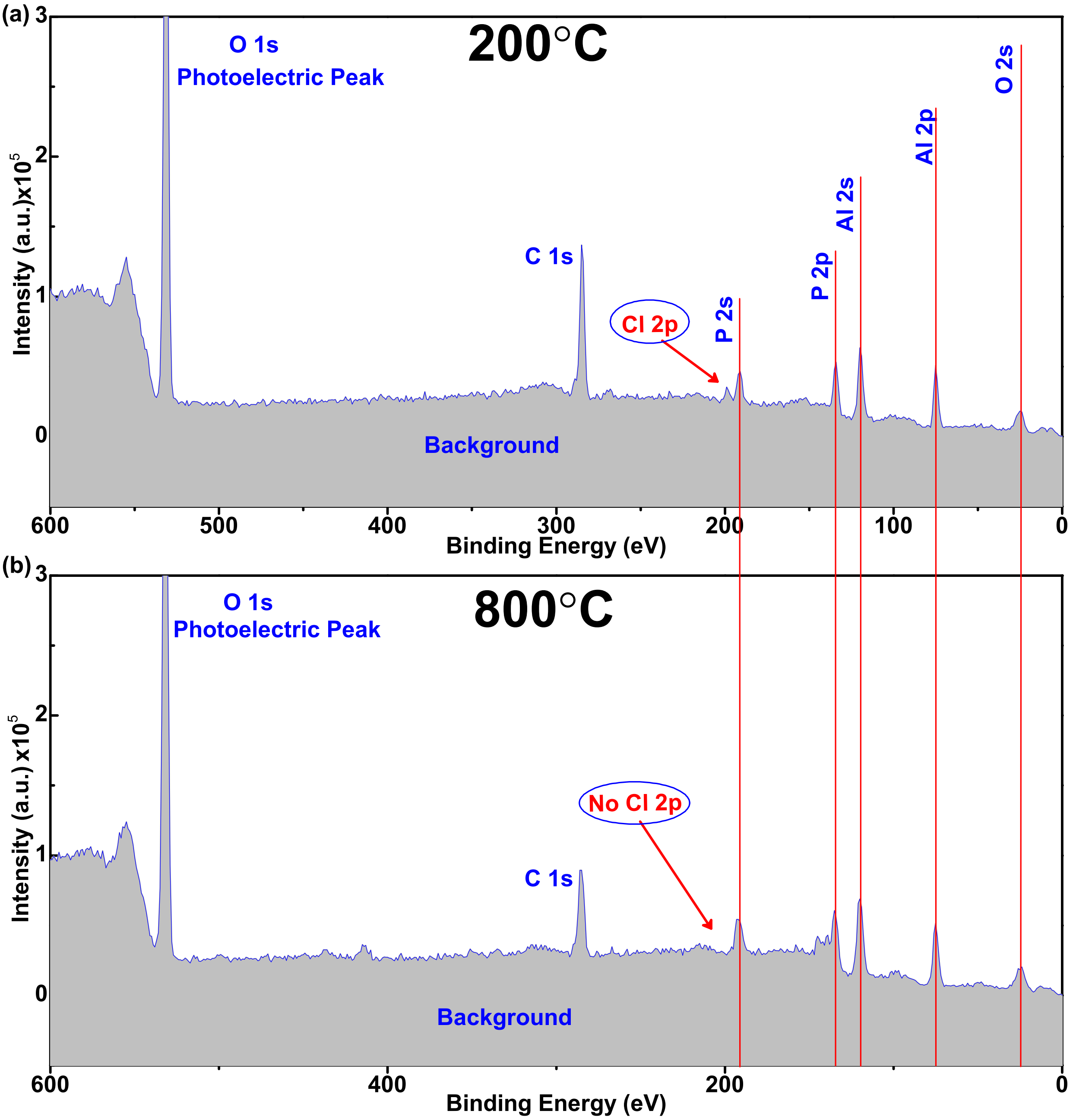

Supplementary Figure 9 | Presence of Cl in survey scan of low temperature processed ALPO : The XPS survey scan spectrum was performed by using 180 eV pass energy with a step 1eV (a) The presence of Cl is shown in ALPO annealed at 200°C. (b) There is no Cl observed in ALPO which is annealed at 800°C. The C 1s peak arises from the film surface and disappears after argon etching.

Supplementary Table−2

Total oxygen and chlorine concentration as a function of annealing temperature

Device Anneal Temp.[°C]	O(%)	Cl(%)
AP[a]	60.14	0.08
200	59.75	0.53
400	57.26	<0.01
600	52.69	<0.01
800	49.57	<0.01
1000	56.88	<0.01

[a] As prepared devices

SUPPLEMENTARY NOTE 10

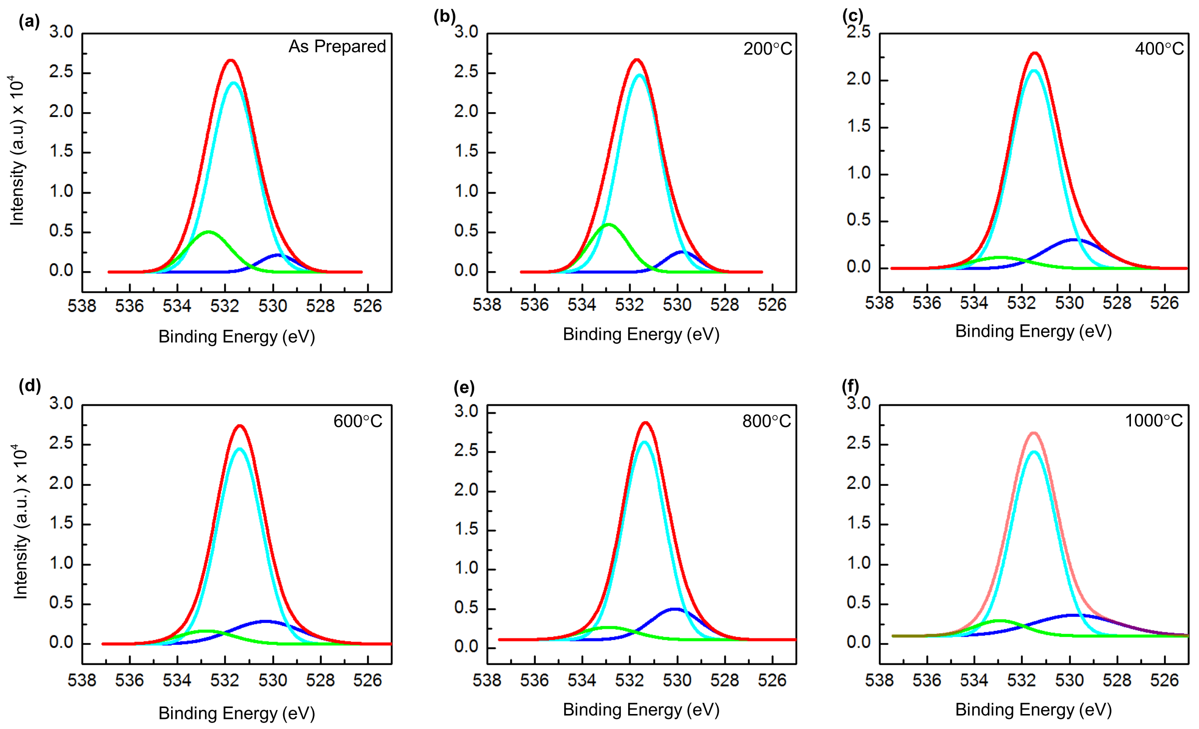

Supplementary Figure 10 | Deconvolution of core oxygen peak: The core level oxygen peak from as−prepared, 200°C, 400°C, 600°C, 800°C and 1000°C heated ALPO film respectively. The oxygen peak(red) in each case is de-convoluted into three components corresponding to oxygen vacancies ($M-O_{vac}$) ∼ green, metal hydroxide (M−OH) ∼ cyan and lattice oxygen (M−O) ∼ blue.

Supplementary Table−3

Quantitative details of temperature dependent changes in core oxygen spectra

Annealing Temperature	Atomic % (From: Survey)	Property of O 1s peak			
		O 1s	M-O	M-OH	M-O_{VAC}
As Prepared	60.1	BAND (eV)	529.8	531.6	532.7
		FWHM (eV)	1.8	2.2	2.1
		AREA (%)	5.5	74.7	15.4
		Atomic %	3.3	44.9	9.3
200°C	59.8	BAND (eV)	529.8	531.6	532.9
		FWHM (eV)	1.7	2.1	1.9
		AREA (%)	5.9	74.3	16.1
		Atomic %	3.5	44.4	9.6
400°C	57.3	BAND (eV)	529.8	531.5	532.7
		FWHM (eV)	2.9	2.3	3
		AREA (%)	14.9	77.3	6.6
		Atomic %	8.5	44.3	3.8
600°C	52.7	BAND (eV)	530.3	531.4	532.8
		FWHM (eV)	3	2.2	3.4
		AREA (%)	15.6	77.2	6.2
		Atomic %	8.2	40.7	3.3
800°C	49.6	BAND (eV)	530.1	531.4	532.9
		FWHM (eV)	2.4	2.1	2.7
		AREA (%)	14	77.2	6.9
		Atomic %	6.9	38.3	3
1000°C	56.9	BAND (eV)	529.8	531.5	532.9
		FWHM (eV)	4.1	2.2	2.4
		AREA (%)	16	75.5	6.9
		Atomic %	9.1	42.6	3.9
